# Correlation between Serum Platelet Count and Cognitive Function in Patients with Atrial Fibrillation: A Cross-Sectional Study

**DOI:** 10.1155/2021/9039610

**Published:** 2021-03-22

**Authors:** Dandan Sun, Quanliang Wang, Jie Kang, Jie Zhou, Ruijuan Qian, Wenqing Wang, Haichen Wang, Qingyun Zhang

**Affiliations:** Department of Cardiology of Affiliated Hospital, Jining Medical University, 89# Guhuai Road, Rencheng District, Jining City 272000, Shandong Province, China

## Abstract

**Background:**

The risk of cognitive impairment in patients with atrial fibrillation is significantly increased. Its occurrence may be related to blood hypercoagulable state and immune inflammatory reaction. Platelets can mediate immune inflammatory response, but there is no evidence about the relationship between platelet count and cognitive function in patients with atrial fibrillation.

**Purpose:**

To explore whether there is a certain correlation between platelet count and cognitive function in patients with atrial fibrillation.

**Methods:**

A cross-sectional study was conducted in a single center in China, including 254 patients with atrial fibrillation. Cognitive function assessment and clinical and laboratory examinations were performed on all participants. After adjusting the related confounding factors, the relationship between platelet count and cognitive function was analyzed.

**Results:**

A total of 254 subjects with an average age of 59.71 ± 11.14 years were included. The average platelet count was 208.15 ± 68.30, and the average score of cognitive function was 19.29 ± 6.78. Result of fully adjusted binary logistic regression showed platelet count was negatively associated with the cognitive function score after adjusting confounders (hazard ratio (HR) = 0.000, 95%CI −0.01, 0.01). A nonlinear relationship was detected between platelet count and the cognitive function score, whose point was 230. The effect sizes and the confidence intervals of the left and right sides of the inflection point were 0.03 (0.01–0.05, *P* for nonlinearity = 0.011) and −0.03 (−0.05–0.00, *P* for nonlinearity = 0.023), respectively.

**Conclusion:**

Platelets have a nonlinear relationship with cognitive function in patients with atrial fibrillation. This finding suggests that, in patients with atrial fibrillation, platelets should be maintained at about 230.

## 1. Introduction

Atrial fibrillation is the most common type of arrhythmia. In people aged 60 to 65, the incidence of atrial fibrillation is less than 1%. But, in people over the age of 80, the incidence of atrial fibrillation is 8% to 10% [1]. Surveys in 10 regions of China show that the prevalence of atrial fibrillation is 0.42% in people aged 35 to 59 years and as high as 1.83% in people over 60 years of age [2]. In 2010, the total number of people with atrial fibrillation in the world was 335 million, which is expected to reach 600 million by 2050 [3].

Atrial fibrillation will seriously affect the prognosis of patients. Studies have shown that the incidence of stroke in patients with atrial fibrillation will increase fivefold. Persistent atrial fibrillation accounts for 35% of all-cause mortality in patients with atrial fibrillation [4]. A 14-year study of 272186 patients with atrial fibrillation showed that the risk of death in women and men with atrial fibrillation was 1.50 and 1.28 times higher than that in the normal population, respectively [5]. Secondly, in recent years, more and more scholars have paid attention to the serious impact of atrial fibrillation on the cognitive function of patients. Many studies have shown that atrial fibrillation will lead to cognitive impairment in patients. The study of Alvaro Alonso [6] has shown that the incidence of dementia and mild cognitive impairment in patients with atrial fibrillation is 40%, and the risk is 2.25 times and 1.28 times higher than that in normal people, respectively. In addition, the study of Lin Y et al. [7] has shown that atrial fibrillation is a risk factor for dementia and cognitive impairment, and this relationship still exists after excluding the effects of stroke.

At present, the mechanism of cognitive impairment in patients with atrial fibrillation is not completely clear. The possible mechanisms include the following points [8–10]: (1) blood hypercoagulable state; (2) the formation of inflammatory factors; (3) low cerebral blood perfusion; and (4) genes and other factors. The blood hypercoagulable state may affect the cognitive function of patients. The renin-angiotensin-aldosterone system is activated, and the neuroendocrine system shows a certain degree of disorder, resulting in vascular endothelial damage, cerebral vasoconstriction, and increased cerebral blood flow resistance, coupled with blood hypercoagulable state, microthrombosis, long-term microthrombotic state, leading to cognitive impairment.

The main functions of platelets are coagulation and hemostasis, repair of damaged blood vessels, protection of the vascular endothelium, participation in endothelial repair, and prevention of atherosclerosis. At the same time, platelets have the function of mediating immune inflammatory response [11]. This study hypothesized that platelets may also affect the cognitive function of patients with atrial fibrillation through this mechanism. At present, there is a lack of evidence of the relationship between platelets and cognitive impairment. Therefore, the purpose of this study is to explore whether there is a certain correlation between platelet count and cognitive function in patients with atrial fibrillation.

## 2. Methods

### 2.1. Study Design

The purpose of this cross-sectional study was to explore the correlation between plasma platelet count and cognitive function in patients with atrial fibrillation. The independent variable of the study was platelet content (continuous variable), and the dependent variable was cognitive function (continuous variable).

### 2.2. Participants

A total of 254 patients with atrial fibrillation were conveniently selected from December 1, 2018, to December 1, 2019, in the Department of Cardiology, Affiliated Hospital of Jining Medical College, Jining City, Shandong Province. Inclusion criteria were age more than 18 years; clinically diagnosed with atrial fibrillation; informed consent to this study; and signed informed consent form. Exclusion criteria were patients with deafness; patients with no basic reading and writing ability; patients with severe illness; and could not cooperate. The diagnosis of atrial fibrillation is based on 12-lead electrocardiogram and 24-hour ambulatory electrocardiogram. According to the onset time of atrial fibrillation, the patients were diagnosed as paroxysmal atrial fibrillation, persistent atrial fibrillation, long-term persistent atrial fibrillation, and permanent atrial fibrillation.

### 2.3. Data Collection

General data of patients were collected, including age, sex, marital status, education, living conditions, smoking, drinking, BMI index, and other indicators. At the same time, the clinical examination and examination results of the patients were collected including hemoglobin (Hb), red blood cell (RBC), prothrombin time (PT), International Normalized Ratio (INR), activated partial thromboplastin time (APTT), D-dimer, free triiodothyronine (FT3), free thyroxine (FT4), thyroid stimulating hormone (TSH), creatinine, urea, uric acid (UA), aspartate transaminase (AST), alanine aminotransferase (ALT), lipoprotein, total cholesterol (TC), triacylglycerol (TG), low-density lipoprotein (LDL), and left ventricular ejection fraction (LVEF) (%). The patients' past history (including hypertension, diabetes, heart failure, coronary heart disease, myocardial infarction, hyperlipidemia, valvular disease, and cerebral infarction) and medication history within 1 month before admission (including aspirin, warfarin, clopidogrel, tegrel, and rivashaban) were collected. This study was approved by the Medical Scientific Research Ethics Committee of the affiliated Hospital of Jining Medical College.

The cognitive function of the patients was evaluated with the Montreal Cognitive function Assessment scale (MoCA), which was sinicized by the Chinese cultural background. It includes the following aspects: (1) short-term memory: learning and memorizing 5 words twice and delayed recall after about 5 minutes (5 points); (2) visual-spatial ability: clock drawing test (3 points) and copying three-dimensional cube graphics (1 point); (3) executive function: modified wiring test B (1 score), language fluency test (1 score), and word similarity test (2 points); (4) attention, computational ability, and working memory: target number recognition (1 score), continuous subtraction test (3 points), and digit span test (2 points); (5) language: common animal naming test (3 points), retelling 2 complex sentences (2 points), and aforementioned language fluency; and (6) orientation: time and place orientation (6 points). If the subjects have less than 12 years of education, we will add 1 point to the total score. The full score of MoCA is 30. The higher the score, the better the cognitive function. The test will take about 10 minutes.

### 2.4. Statistical Analysis

The continuous variables were presented in two forms. The K-S method was used to conduct the normality test. In the first form, continuous variables with normal distribution were expressed as mean ± standard deviation. In the second form, continuous variables with skewed distribution were presented as median (min, max). The categorical variables were expressed as frequency or percentage. The chi-square (categorical variables), one-way analysis of variance (normal distribution), or Kruskal–Wallis H tests (skewed distribution) were used for the differences among different platelet groups (trisection based on platelets data). The data analysis was based on three criteria: (1) the relationship between platelet count and cognitive function (linear or nonlinear), (2) the factors modifying or interfering with the relationship between platelet count and cognitive function, and (3) the interference factors or the true relationship between platelet count and cognitive function after the stratified analysis. Therefore, data analysis was summarized in three steps. Step 1: univariate and multivariate binary logistic regression analyses were employed. Two models were constructed: model 1, a crude model with no covariates adjusted; model 2, adjusted only for sociodemographic data; and model 3, model 2 + other covariates presented in Table 1. Step 2: a generalized additive model and smooth curve fitting (penalized spline method) were employed to address the nonlinearity of platelets and cognitive function. If nonlinearity was detected, first, the inflection point was determined using the recursive algorithm and then a two-piecewise binary logistic regression was constructed on both sides of the inflection point. Determining which model was more suitable for fitting the correlation between target independent and dependent variables was based mainly on the *P* value of the log likelihood ratio test. The continuous variable was first converted into a quartile categorical variable, and then, an interaction test was performed. The tests for effect modification for subgroup indicators were followed by the likelihood ratio test. A sensitivity analysis was conducted to ensure the robustness of data analysis [12]. The platelet count was converted into a categorical variable, and the *P* value was calculated for the trend. The purpose was to verify the results of platelet count as the continuous variable and observe the possibility of nonlinearity. All the analyses were performed with the statistical software packages *R* (http://www.R-project.org, The *R* Foundation) and EmpowerStats (http://www.empowerstats.com, *X* & *Y* Solutions, Inc, MA, USA). *P* values less than 0.05 (two-sided) were considered statistically significant.

## 3. Results

### 3.1. Clinical and Laboratory Characteristics of the Subjects

We divided the study population into three groups according to platelet count (T1: <172 *∗* 10^9/L; T2: 172–225 *∗* 10^9/L; T3: >225 *∗* 10^9/L). The clinical and laboratory characteristics are described in Table 1. A total of 254 subjects with an average age of 59.71 ± 11.14 years were included. A total of 141 (55.51%) male patients with atrial fibrillation were included in all the subjects. The average platelet count was 208.15 ± 68.30. The average score of cognitive function was 19.29 ± 6.78. One hundred and forty-three (56.30%) patients had heart failure, and 136 (53.54%) patients had hypertension. There were 76 (29.92%) patients with paroxysmal atrial fibrillation, 124 (48.82%) patients with persistent atrial fibrillation, and 24 (9.45%) patients with long-term persistent atrial fibrillation.

### 3.2. Factors Correlated with Cognitive Function in the Subjects

Univariate linear regression analysis was performed to determine the relationships between clinical parameters and cognitive function. As shown in Table 2, variables significantly related to cognitive function scores include age, sex, education, smoking and drinking, BMI, Hb, RBC, D-dimer, urea, heart failure, hyperlipidemia, and valvular disease (*P* < 0.05). No significant correlation was observed between variables and cognitive function such as marital status, living condition, PT, INR, APTT, FT3, FT4, creatinine, UA, AST, ALT, lipoprotein, TC, TG, HDL, LDL, LVEF, hypertension, diabetes mellitus, coronary artery disease, myocardial infarction, cerebral infarction history, warfarin, clopidogrel, ticagrelor, rivashaban, type of AF, and duration of AF (*P* > 0.05).

### 3.3. Independent Correlation between Platelet Count and Cognitive Function by Multivariate Piecewise Linear Regression

**Figure 1:**
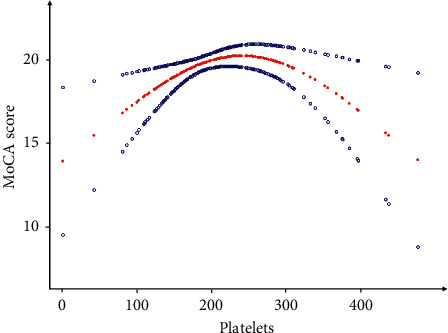
Association between platelets and cognitive function of atrial fibrillation. A threshold and a nonlinear relationship between platelet count and the MoCA score were found in a generalized additive model. The solid red line represents the smooth curve fit between variables. Blue bands represent the 95% confidence interval from the fit. Models were adjusted for age; sex; BMI; educational level; smoking; alcohol consumption; valvular disease; hypertension; diabetes mellitus; heart failure; coronary artery disease; myocardial infarction; hyperlipidemia; cerebral infarction history; aspirin; warfarin; clopidogrel; rivashaban; type of AF; duration of AF; Hb; RBC; PT; INR; APTT; D-dimer; FT3; FT4; TSH; creatinine; urea; UA; AST; ALT; lipoprotein; TC; TG; HDL; LDL; and LVEF.

As shown in Figure 1, smooth curve fitting is carried out after adjusting the possible confounding factors, including age, sex, marital status, educational level, and living condition. There was a nonlinear relationship between the cognitive function score and platelet count in patients with atrial fibrillation, and the resultant curve showed two-stage changes and a critical point. When the platelet count is greater than the critical point, the platelet count is negatively correlated with the cognitive function score, and when the platelet count is less than the critical point, the platelet count is positively correlated with the cognitive function score. As shown in Table 3, the threshold effect is further analyzed by curve fitting. Because the *P* for the log likelihood ratio test was less than 0.05, we chose two-piecewise binary logistic regression for fitting the association between platelet count and cognitive function because it can accurately represent the relationship. The data show that the inflection point of platelet count is 230. When the platelet count is greater than 230, the level of cognitive function decreases with the increase of platelets (*β* = 0.03, 95%CI 0.01–0.95, *P* = 0.023). When the platelet count is less than 230, with the increase of platelet count, the level of cognitive function tends to increase, with a significant statistical significance (*β* = −0.03, 95%CI −0.05–0.00, *P* = 0.011) (Table 4).

## 4. Discussion

In our study, there was a nonlinear relationship between platelet count and cognitive function in patients with atrial fibrillation, and the turning point was 230. When the platelet count was less than 230, the cognitive function score of patients with atrial fibrillation increased significantly with the increase of platelet count. When the platelet count was greater than 230, the cognitive function of patients with atrial fibrillation decreased significantly with the increase of platelet count.

The results of this study revealed the effect of platelet count on cognitive function in patients with atrial fibrillation, which proved the relationship between the platelet and brain from a clinical point of view, and found the threshold point of platelet count. In previous studies [13–17], most of the studies on the relationship between platelets and cognitive function focused on the basic research of the pathophysiological mechanism. Atrial fibrillation has been considered as one of the risk factors of cognitive impairment in patients [7, 9, 18–21], but its mechanism is not completely clear. The mechanisms may include blood hypercoagulable state, immunoinflammatory response, low cerebral blood perfusion, genes, and other factors. Among them, platelets may affect the cognitive function of patients in the following ways [22]: (1) platelets can mediate immune response and secrete proinflammatory mediators [23–25]; (2) platelets promote nerve tissue regeneration and reduce neuronal apoptosis [26–28]; and (3) platelet alpha granules carry a series of bioactive molecules to promote neurogenesis and angiogenesis [29, 30]. Arrhythmia is associated with increased platelet reactivity. Compared with patients with sinus rhythm, platelet reactivity in patients with atrial fibrillation is significantly higher, which is closely related to the development of thrombus [31]. Platelets can respond to the circulatory environment of atrial fibrillation by changing transcriptional products and prethrombotic states, and platelets may have molecular “reprogramming” caused by blood flow interference of atrial fibrillation [32].

Platelets have been considered as a marker for the diagnosis of dementia. Studies have shown that platelet APP ratio (representing the percentage of 120–130 kDa to 110 kDa isoforms of the amyloid precursor protein) is reduced in patients with mild cognitive impairment (MCI) and Alzheimer's disease (AD) [33]. Oluwatomi et al. [34] discussed platelets as biomarkers of dementia and analyzed their potential as clinical biomarkers of various subtypes of dementia. The platelet protein biomarkers that have been studied to diagnose dementia (especially Alzheimer's disease) include amyloid protein precursor (A *β* PP), A *β* PP secretase (BACE1 and *β* 10), *α*-synuclein, tau protein, 5-hydroxytryptamine, cholesterol, phospholipase, aggregin, immunoglobulin, surface receptor, MAO-B, and coated platelet. Among them, platelet tau, A-*β*-PP (especially coated platelets) and secreted ADAM-10 and BACE1 are the most promising for clinical diagnosis of dementia. The changes of mean platelet volume and other factors may play a very special role in the diagnosis and prognosis of dementia. The results of our study found that the positive correlation between cognitive function and platelets exists only when the platelet count is less than 230. This result suggests that, for patients with atrial fibrillation, when the platelet count exceeds a certain number, brain function may be affected through other inhibitory mechanisms. Therefore, our research also provides a direction for future basic and clinical research. We can predict the possibility of cognitive impairment based on platelet count. In addition, platelets can be used as biomarkers to build a prediction model. Traditional Chinese medicine with fewer side effects can be used to maintain the platelet count in patients with atrial fibrillation at about 230, such as Xuesheng capsule, rise platelet capsule, and compound soap alum pills. For patients with high platelet count and high risk of thrombosis, antiplatelet aggregation drugs such as aspirin and clopidogrel can be used.

In addition, our study also found that the cognitive function of patients with atrial fibrillation is affected by age, sex, education, smoking, drinking, body mass index, hemoglobin, urea, D-dimer, heart failure, vascular disease, and other factors. This is consistent with some previous studies [35–45].

The strengths and innovations of this study include the following: (1) we explored and described the relationship between platelet count and cognitive function in patients with atrial fibrillation from a clinical point of view; (2) we found the turning point of the effect of platelets on cognitive function through a curve fitting model, which can provide a certain reference value for clinical treatment; and (3) the cognitive function of Chinese patients with atrial fibrillation was evaluated, and some risk factors were identified. However, this study still has some limitations: (1) the sample size of this study is small, and only 254 patients with atrial fibrillation are evaluated and collected; (2) this study is a single-center study. Only patients with atrial fibrillation in the Affiliated Hospital of Jining Medical College were studied. (3) This study is a cross-sectional study, and there is no follow-up of the patients. In the future, the sample size can be expanded, data collection can be carried out in more hospitals in multiple regions, and patients with atrial fibrillation can be followed up for a long time to study the effect of platelet count on the long-term outcome of patients with atrial fibrillation. In the future, we can also explore the correlation between platelet count and cognitive function in patients with nonatrial fibrillation.

## 5. Conclusions

In conclusion, this study describes the nonlinear relationship between cognitive function and platelet count in patients with atrial fibrillation after adjusting for confounding factors. This finding suggests that, in patients with atrial fibrillation, platelets should be maintained at about 230, and too high or too low will affect cognitive function to a certain extent.

## Figures and Tables

**Table 1:** Baseline characteristics of participants.

Platelets (*∗*10^9/L)	T1 ＜172	T2 172–225	T3 ＞225	*P* value
*N*	83	85	86	
Age (year)	68.46 ± 11.32	67.56 ± 10.08	66.10 ± 9.92	0.165
Sex, *n* (%)				0.006
Female	56 (67.47)	48 (56.47)	37 (43.02)	
Male	27 (32.53)	37 (43.53)	49 (56.98)	
Degree of education, *n* (%)				0.240
Illiterate	24 (28.92)	30 (35.29)	30 (34.88)	
Primary and junior high school	43 (51.81)	36 (42.35)	40 (46.51)	
High school	10 (12.05)	9 (10.59)	14 (16.28)	
Undergraduate and above	6 (7.23)	10 (11.76)	2 (2.33)	
Smoking or not, *n* (%)				0.185
Nonsmoker	42 (50.60)	48 (56.47)	57 (66.28)	
Current smoker	19 (22.89)	22 (25.88)	17 (19.77)	
Quit	22 (26.51)	15 (17.65)	12 (13.95)	
Alcohol consumption, *n* (%)				0.137
Nondrinker	51 (61.45)	61 (71.76)	67 (77.91)	
Current drinker	20 (24.10)	16 (18.82)	15 (17.44)	
Quit	12 (14.46)	8 (9.41)	4 (4.65)	
BMI (kg/m^2^)	24.64 ± 4.32	26.06 ± 4.47	25.23 ± 3.98	0.132
Hb (g/L)	131.89 ± 21.09	138.41 ± 17.84	134.20 ± 19.38	0.176
RBC (*∗*10^12/L)	4.20 ± 0.67	4.51 ± 0.63	4.52 ± 0.66	0.003
PT (s)	15.70 ± 8.52	14.98 ± 10.01	13.53 ± 6.75	0.008
INR	1.42 ± 0.80	1.33 ± 0.88	1.20 ± 0.60	0.007
APTT (s)	33.02 ± 8.04	32.95 ± 8.18	31.34 ± 4.45	0.209
D-dimer (mg/L)	1.21 ± 2.24	0.87 ± 1.53	0.81 ± 0.91	0.068
FT3 (pmol/L)	4.66 ± 3.82	5.00 ± 2.69	4.76 ± 3.42	0.025
FT4 (pmol/L)	19.38 ± 10.61	18.12 ± 6.34	19.87 ± 13.79	0.554
TSH (mIU/L)	3.59 ± 4.48	3.00 ± 3.16	2.79 ± 2.14	0.597
Creatinine (umol/L)	81.31 ± 28.80	71.88 ± 24.03	71.99 ± 37.10	0.007
Urea (mmol/L)	8.42 ± 4.07	6.75 ± 3.37	6.87 ± 3.64	<0.001
UA (umol/L)	371.50 (30.00–745.00)	344.00 (192.00–1096.00)	345.00 (163.00–778.00)	0.044
AST (U/L)	22.00 (6.60–618.00)	19.00 (6.30–3319.00)	20.00 (5.20–1847.00)	0.248
ALT (U/L)	16.90 (5.50–258.70)	21.00 (7.20–1373.30)	19.30 (3.70–1095.90)	0.502
Lipoprotein (mg/L)	172.50 (3.00–1015.00)	164.50 (3.00–804.00)	214.00 (3.00–1091.00)	0.347
TC (mmol/L)	3.46 ± 0.91	3.87 ± 0.90	3.95 ± 1.01	0.002
TG (mmol/L)	0.99 ± 0.59	1.33 ± 0.68	1.27 ± 0.60	<0.001
HDL (mmol/L)	1.03 ± 0.26	1.08 ± 0.26	1.08 ± 0.30	0.609
LDL (mmol/L)	2.02 ± 0.70	2.28 ± 0.75	2.37 ± 0.74	0.012
LVEF (%)	46.96 ± 13.86	50.27 ± 11.29	52.65 ± 10.45	0.015
MoCA score	18.87 ± 6.58	19.58 ± 6.43	19.14 ± 7.42	0.800
Hypertension, *n* (%)				0.224
No	45 (54.22)	36 (42.35)	37 (43.02)	
Yes	38 (45.78)	49 (57.65)	49 (56.98)	
Diabetes mellitus, *n* (%)				0.479
No	66 (79.52)	62 (72.94)	62 (72.09)	
Yes	17 (20.48)	23 (27.06)	24 (27.91)	
Heart failure, *n* (%)				0.009
No	25 (30.12)	44 (51.76)	42 (48.84)	
Yes	58 (69.88)	41 (48.24)	44 (51.16)	
Coronary artery disease, *n* (%)				0.003
No	26 (31.33)	13 (15.29)	10 (11.63)	
Yes	57 (68.67)	72 (84.71)	76 (88.37)	
Myocardial infarction, *n* (%)				0.338
No	77 (92.77)	75 (88.24)	81 (94.19)	
Yes	6 (7.23)	10 (11.76)	5 (5.81)	
Hyperlipidemia, *n* (%)				0.375
No	81 (97.59)	83 (97.65)	81 (94.19)	
Yes	2 (2.41%)	2 (2.35)	5 (5.81)	
Valvular disease, *n* (%)				0.050
No	47 (56.63)	62 (72.94)	61 (70.93)	
Yes	36 (43.37)	23 (27.06)	25 (29.07)	
Cerebral infarction history, *n* (%)				0.304
No	68 (81.93)	68 (80.00)	76 (88.37)	
Yes	15 (18.07)	17 (20.00)	10 (11.63)	
Aspirin, *n* (%)				0.746
No	56 (67.47)	53 (62.35)	54 (62.79)	
Yes	27 (32.53)	32 (37.65)	32 (37.21)	
Warfarin, *n* (%)				0.295
No	70 (84.34)	73 (85.88)	79 (91.86)	
Yes	13 (15.66)	12 (14.12)	7 (8.14)	
Clopidogrel, *n* (%)				0.573
No	79 (95.18)	78 (91.76)	78 (90.70)	
Yes	4 (4.82)	7 (8.24)	8 (9.30)	
Rivashaban, *n* (%)				0.293
No	77 (92.77)	79 (92.94)	84 (97.67)	
Yes	6 (7.23)	6 (7.06)	2 (2.33)	
Type of AF, *n* (%)				0.269
Paroxysmal AF	21 (25.30)	32 (37.65)	23 (26.74)	
Persistent AF	45 (54.22)	32 (37.65)	47 (54.65)	
Long-term persistent AF	7 (8.43)	11 (12.94)	6 (6.98)	
Permanent	10 (12.05)	10 (11.76)	10 (11.63)	
Duration of AF, *n* (%)				0.323
≤1 year	28 (33.73)	31 (36.47)	43 (50.00)	
1–5 years	29 (34.94)	24 (28.24)	23 (26.74)	
6–10 years	10 (12.05)	13 (15.29)	9 (10.47)	
＞10 years	16 (19.28)	17 (20.00)	11(12.79)	

Hb, hemoglobin; RBC, red blood cell; PT, prothrombin time; INR, International Normalized Ratio; APTT, activated partial thromboplastin time; FT3, free triiodothyronine; FT4, free thyroxine; TSH, thyroid-stimulating hormone; UA, uric acid; AST, aspartate transaminase; ALT, alanine aminotransferase; TC, total cholesterol; TG, triacylglycerol; HDL-C, high-density lipoprotein cholesterol; LDL-C, low-density lipoprotein cholesterol. *P* < 0.05 is considered to be statistically significant.

**Table 2:** Univariate analysis for cognitive function of atrial fibrillation patients.

Covariate	Statistics	*β* (95%CI)	*P* value
Age (year)	59.71 ± 11.14	1.03 (1.02–1.04)	<0.001
Sex, *n* (%)			
Male	141 (55.51%)	Reference	<0.001
Female	113 (44.49%)	−4.91 (−6.46, −3.36)	
Degree of education, *n* (%)			
Illiterate	84 (33.07%)	Reference	
Primary and junior high school	119 (46.85%)	6.82 (5.32, 8.31)	<0.001
High school	33 (12.99%)	10.79 (8.62, 12.96)	<0.001
Undergraduate and above	18 (7.09%)	11.81 (9.19, 14.44)	<0.001
Smoking or not, *n* (%)			
Nonsmoker	147 (57.87%)	Reference	
Current smoker	58 (22.83%)	3.89 (1.91, 5.87)	<0.001
Quit	49 (19.29%)	3.67 (1.57, 5.77)	<0.001
Alcohol consumption, *n* (%)			
Nondrinker	179 (70.47%)	Reference	
Current drinker	51 (20.08%)	4.96 (2.98, 6.95)	<0.001
Quit	24 (9.45%)	2.82 (0.06, 5.58)	0.047
PLT	208.1 ± 68.2	0.00 (−0.01, 0.01)	0.575
BMI	25.33 ± 4.27	0.35 (0.16, 0.54)	<0.001
Hb (g/L)	134.85 ± 19.58	0.08 (0.03, 0.12)	＜0.001
RBC (*∗*10^12/L)	4.41 ± 0.67	1.44 (0.19, 2.69)	0.024
PT (s)	12.15 (10.00–66.00)	−0.07 (−0.17, 0.03)	0.154
INR	1.32 ± 0.77	−0.87 (−1.94, 0.21)	0.115
APTT (s)	32.53 ± 7.26	0.10 (−0.01, 0.21)	0.084
D-dimer (*∗*10^12/L)	0.55 (0.04–15.37)	−1.00 (−1.51, −0.50)	＜0.001
FT3 (pmol/L)	4.31 (0.94–31.80)	0.08 (−0.18, 0.35)	0.546
FT4 (pmol/L)	17.60 (3.72–100.00)	−0.05 (−0.14, 0.03)	0.196
TSH (mIU/L)	2.40 (0.01–33.30)	0.01 (−0.25, 0.27)	0.945
Creatinine (umol/L)	74.92 ± 30.66	−0.00 (−0.03, 0.02)	0.778
Urea (mmol/L)	7.31 ± 3.74	−0.24 (−0.47, −0.02)	0.034
UA (umol/L)	376.68 ± 129.03	0.00 (−0.01, 0.01)	0.854
AST (U/L)	20.00 (5.20–3319.00)	−0.00 (−0.00, 0.00)	0.397
ALT (U/L)	19.90 (3.70–1373.30)	−0.00 (−0.01, 0.01)	0.660
Lipoprotein (mg/L)	188.00 (3.00–1091.00)	0.00 (−0.00, 0.00)	0.617
TC (mmol/L)	3.77 ± 0.96	−0.49 (−1.38, 0.40)	0.280
TG (mmol/L)	1.20 ± 0.64	0.74 (−0.59, 2.08)	0.275
HDL (mmol/L)	1.07 ± 0.28	−0.66 (−3.74, 2.43)	0.678
LDL (mmol/L)	2.23 ± 0.74	−0.59 (−1.74, 0.56)	0.312
LVEF (%)	50.09 ± 12.05	0.03 (−0.04, 0.10)	0.354
Hypertension, *n* (%)			
No	118 (46.46%)	Reference	
Yes	136 (53.54%)	−0.51 (−2.16, 1.14)	0.545
Diabetes mellitus, *n* (%)			
No	190 (74.80%)	Reference	
Yes	64 (25.20%)	0.54 (−1.37, 2.44)	0.580
Heart failure, *n* (%)			
No	111 (43.70%)	Reference	
Yes	143 (56.30%)	−3.45 (−5.05, −1.84)	<0.001
Coronary artery disease, *n* (%)			
No	49 (19.29%)	Reference	
Yes	205 (80.71%)	−0.46 (−2.52, 1.61)	0.665
Myocardial infarction, *n* (%)			
No	233 (91.73%)	Reference	0.253
Yes	21 (8.27%)	−1.76 (−4.78, 1.26)	
Hyperlipidemia, *n* (%)			
No	245 (96.46%)	Reference	0.026
Yes	9 (3.54%)	5.11 (0.64, 9.58)	
Valvular disease, *n* (%)			
No	170 (66.93%)	Reference	
Yes	84 (33.07%)	−2.32 (−4.04, −0.59)	0.009
Cerebral infarction history, *n* (%)			
No	212 (83.46%)	Reference	0.600
Yes	42 (16.54%)	−0.60 (−2.84, 1.64)	
Aspirin, *n* (%)			
No	163 (64.17%)	Reference	0.082
Yes	91 (35.83%)	1.53 (−0.18, 3.24)	
Warfarin, *n* (%)			
No	222 (87.40%)	Reference	0.712
Yes	32 (12.60%)	0.47 (−2.01, 2.95)	
Clopidogrel, *n* (%)			
No	235 (92.52%)	Reference	0.765
Yes	19 (7.48%)	0.48 (−2.69, 3.65)	
Rivashaban, *n* (%)			
No	240 (94.49%)	Reference	0.676
Yes	14 (5.51%)	0.76 (−2.78, 4.29)	
Type of AF, *n* (%)			
Paroxysmal AF	76 (29.92%)	Reference	
Persistent AF	124 (48.82%)	−1.61 (−3.51, 0.30)	0.099
Long-term persistent AF	24 (9.45%)	−0.01 (−3.11, 3.08)	0.993
Permanent	30 (11.81%)	−0.59 (−3.40, 2.22)	0.680
Duration of AF, *n* (%)			
≤1 year	102 (40.16%)	Reference	
1–5 years	76 (29.92%)	0.20 (−1.79, 2.20)	0.842
6–10 years	32 (12.60%)	0.67 (−2.04, 3.37)	0.629
＞10 years	44 (17.32%)	−0.51 (−2.92, 1.89)	0.676

Hb, hemoglobin; RBC, red blood cell; PT, prothrombin time; INR, International Normalized Ratio; APTT, activated partial thromboplastin time; FT3, free triiodothyronine; FT4, free thyroxine; TSH, thyroid-stimulating hormone; UA, uric acid; AST, aspartate transaminase; ALT, alanine aminotransferase; TC, total cholesterol; TG, triacylglycerol; HDL-C, high-density lipoprotein cholesterol; LDL-C, low-density lipoprotein cholesterol; CI, confidence interval. *P* < 0.05 is considered to be statistically significant.

**Table 3:** Relationship between platelet count and cognitive function in different models.

Variable	Crude model	Adjust I	Adjust II
Platelets	0.00 (−0.01, 0.01) 0.6716	0.00 (−0.01, 0.01) 0.5349	0.00 (−0.01, 0.01) 0.7866
Platelets			
T1	Reference	Reference	Reference
T2	0.71 (−1.36, 2.77) 0.5014	1.00 (−0.82, 2.83) 0.2813	1.25 (−0.75, 3.25) 0.2210
T3	0.27 (−1.79, 2.33) 0.7958	0.85 (−1.01, 2.71) 0.3707	1.32 (−0.87, 3.51) 0.2397
*P* for trend	0.13 (−0.90, 1.16) 0.8005	0.42 (−0.51, 1.35) 0.3725	0.64 (−0.45, 1.73) 0.2532

Nonadjusted model adjusted for none. Adjust I model adjusted for age and sex. Adjust II model adjusted for age and Sex; BMI; educational level; smoking; alcohol consumption; valvular disease; hypertension; diabetes mellitus; heart failure; coronary artery disease; myocardial infarction; hyperlipidemia; cerebral infarction history; aspirin; warfarin; clopidogrel; rivashaban; type of AF; duration of AF; Hb; RBC; PT; INR; APTT; D-dimer; FT3; FT4; TSH; creatinine; urea; UA; AST; ALT; lipoprotein; TC; TG; HDL; LDL; LVEF.

**Table 4:** Results of platelet count and cognitive function using two-piecewise linear regression.

Inflection point of platelets	Effect size	95% CI	*P* value
＜230	0.03	0.01–0.05	0.011
≥230	−0.03	−0.05–0.00	0.023

Effect: cognitive function; cause: platelet count. Adjusted for age and sex; BMI; educational level; smoking; alcohol consumption; valvular disease; hypertension; diabetes mellitus; heart failure; coronary artery disease; myocardial infarction; hyperlipidemia; cerebral infarction history; aspirin; warfarin; clopidogrel; rivashaban; type of AF; duration of AF; Hb; RBC; PT; INR; APTT; D-dimer; FT3; FT4; TSH; creatinine; urea; UA; AST; ALT; lipoprotein; TC; TG; HDL; LDL; LVEF.

## Data Availability

The data used to support the findings of this study are available from the corresponding author upon request.
